# Age varying polygenic effects on alcohol use in African Americans and European Americans from adolescence to adulthood

**DOI:** 10.1038/s41598-021-01923-x

**Published:** 2021-11-17

**Authors:** Kit K. Elam, Thao Ha, Zoe Neale, Fazil Aliev, Danielle Dick, Kathryn Lemery-Chalfant

**Affiliations:** 1 Department of Applied Health Science, Indiana University, 1025 E. 7th St., Suite 116, Bloomington, IN 47405 USA; 2 Department of Psychology, Arizona State University, Tempe, USA; 3 Department of Psychology, Virgina Commonwealth University, Richmond, USA

**Keywords:** Behavioural genetics, Human behaviour

## Abstract

Genetic effects on alcohol use can vary over time but are often examined using longitudinal models that predict a distal outcome at a single time point. The vast majority of these studies predominately examine effects using White, European American (EA) samples or examine the etiology of genetic variants identified from EA samples in other racial/ethnic populations, leading to inconclusive findings about genetic effects on alcohol use. The current study examined how genetic influences on alcohol use varied by age across a 15 year period within a diverse ethnic/racial sample of adolescents. Using a multi-ethnic approach, polygenic risk scores were created for African American (AA, *n* = 192) and EA samples (*n* = 271) based on racially/ethnically aligned genome wide association studies. Age-varying associations between polygenic scores and alcohol use were examined from age 16 to 30 using time-varying effect models separately for AA and EA samples. Polygenic risk for alcohol use was found to be associated with alcohol use from age 22–27 in the AA sample and from age 24.50 to 29 in the EA sample. Results are discussed relative to the intersection of alcohol use and developmental genetic effects in diverse populations.

## Introduction

Alcohol use progresses non-linearly across development, typically starting in early adolescence, peaking in early adulthood, and then declining across adulthood^1^. Similarly, genetic and environmental effects on alcohol use can vary non-linearly across adolescence and early adulthood^2, 3^. Large scale gene identification efforts, such as genome wide association studies (GWAS), are advancing our understanding of alcohol use outcomes but often collapse across age so do not address developmental genetic effects. Further, the majority of genetics research on alcohol use has utilized White, European American (EA) samples or has examined genetic variants identified in samples of European descent in different ethnic populations ^4^. These considerations significantly limit understanding of developmental genetic influences underlying alcohol use in diverse populations, which in turn has important implications for identifying risk factors and prevention efforts that are effective across ethnic groups and developmental periods^4, 5^.

The present study addresses these gaps by investigating the developmental effect of genetic predisposition for alcohol use in predicting alcohol use from adolescence to adulthood using an innovative time-varying effect modeling approach. This was investigated using polygenic risk scores (PRS) created for African American (AA) and EA samples based on racially/ethnically aligned GWAS.

### Developmental patterns of alcohol use

Alcohol use typically begins in adolescence and peaks in the early 20’s, which is followed by a general decline in use across adulthood^1, 6^. Within this developmental pattern of alcohol use, differences can exist for AA and EA populations. On average, AAs compared to EAs report lower rates of alcohol misuse, higher abstinence, and less frequent alcohol consumption during adolescence and young adulthood^7, 8^. However, AAs are subject to higher rates of some contextual risk factors including discrimination, residential segregation, and limited access to adequate health resources which can increase risk for developing alcohol use problems^5^. These differences in use and contextual risk factors makes identifying etiological influences underlying alcohol use in AA populations a complex and important endeavor. It should be noted that typical developmental patterns of alcohol use differ from those of alcohol abuse, dependence and disorder^9^, and genetic effects on alcohol use differ from effects on more severe alcohol phenotypes^10, 11^ . The present study focuses on developmental genetic effects on alcohol use.

### Genetic effects on alcohol use

Large scale GWAS are advancing efforts to understand genetic variants involved in alcohol use behaviors, the results of which can be leveraged to create polygenic risk scores (PRS) that aggregate very small effects across multiple single nucleotide polymorphisms (SNPs). For example, GWAS-based PRSs of alcohol use have shown associations with alcohol use^12, 13^. A greater number of studies have demonstrated associations between PRSs for alcohol use problems, dependence, and diagnosis and more problematic alcohol use outcomes^14–17^. A recent study by Johnson et al.^12^, found PRS for alcohol consumption and problem use to predict both alcohol use and more severe indices of alcohol use across multiple samples but was examined only for adults of European descent. Additionally, alcohol outcomes were examined at a single distal time point reinforcing the longitudinal salience of genetic effects but limiting developmental interpretations of results. Few studies exist that include longitudinal measurement of alcohol use to enable estimation of developmental change in genetic effects. One study by Li et al.^18^, found a PRS for alcohol dependence predicted trajectories of heavy episodic drinking from age 15.5 to 21.5, but not later use. Further, this study leveraged a piecewise growth model to index early and later trajectories of drinking but this constrains trajectories to a predefined shape whereas genetic effects may be non-linear. Collectively, the majority of literature examining developmental genetic effects on alcohol use have examined alcohol outcomes at a single distal time point or have estimated linear trajectories constrained to a predefined shape, yet evidence from genetic studies indicates that genetic effects can vary non-linearly with age^11, 19^.

One way to address these limitations is to examine age-varying effects. Time-varying effect models (TVEM) are unique in that they model the strength of associations as a variable function of age with no assumption of the shape of these associations^20^. Russell et al.^21^, used TVEM to examine effects of the GABRA2 gene and a preventive intervention on alcohol misuse across ages 11 to 20. This study found unique developmental effects in which genetic influences on past-month alcohol misuse began at age 12 and increased nonlinearly, peaking at age 18–19, then decreased. Also using TVEM, Elam et al.^22^ examined associations between a PRS for aggression and behavioral aggression across early and middle childhood. This study found genetic effects on aggression varied non-linearly with age, illustrating the utility of using TVEM models to understand developmental genetic effects. However, no study has examined the age-varying developmental effects of a PRS for alcohol use on alcohol outcomes using TVEM.

## Genetic effects on alcohol use in diverse populations

Genetics research on alcohol use has primarily been conducted in EA populations leading to severe underrepresentation of diverse racial/ethnic populations^4^. Broadly, twin and molecular genetic research specifically investigating genetic influences in both AA and EA groups have found partial convergence in genetic influences on alcohol use but also unique effects within each group^23–25^. Independent genetic effects are also supported by research showing that SNPs identified from GWASs in one racial/ethnic population do not necessarily show the same patterns of association in other racial/ethnic populations as diversity in genetic ancestry (e.g., prevalence of SNPs, allele frequency, and direction of allelic effect), and variation in environments (e.g., discrimination) can vary widely across different racial/ethnic populations^26, 27^. It is also important to consider population stratification, when differences in population-level characteristics are associated with variation in genetic ancestry, and admixture, when individuals have mixed genetic ancestry. Population stratification and admixture can be addressed by the use of genetic ancestry principal components^28^. These methodological considerations are exacerbated by the fact that GWAS studies have primarily relied on EA populations, and resulting PRS are often tested in other racial/ethnic populations, resulting in significant limitations in the ability for genetic research findings to benefit all populations.

Recent approaches help to address some of these considerations via the formation of multi-ethnic PRSs. The purpose of a multi-ethnic PRS is to improve predictive accuracy of polygenic scores in diverse populations. One approach is to leverage large GWAS data for a specific outcome when available in multiple discovery racial/ethnic populations that are aligned with those in secondary samples such that discovery weights can be leveraged from each separate GWAS to create PRSs within each of the aligned samples. We took this approach and used summary statistics from a multi-ethnic GWAS on alcohol consumption performed separately in EA (N = 200,680) and AA (N = 56,495) individuals as part of the Million Veteran Program (MVP) to create separate PRSs for each ancestry using weights from the respective GWAS^29^. Given the novelty of this approach, replication of this and similar designs should be pursued as part of future studies.

### The present study

In the current study we used a non-linear longitudinal method, TVEM, to examine genetic effects on alcohol use in a diverse sample across adolescence and adulthood. Leveraging summary statistics from a recent multi-ethnic discovery GWAS of alcohol consumption^29^, we used a multi-ethnic PRS approach to calculate separate polygenic scores for EA and AA participants in a target sample. Using TVEM, PRSs for alcohol consumption were examined as predictors of age-varying alcohol use from adolescence to adulthood, separately for AA and EA participants. In particular, we hypothesize that polygenic risk for alcohol use would predict alcohol use in both AA and EA individuals during respective developmental periods of elevated alcohol use.

## Methods

### Participants

Participants were from the Project Alliance 1 study (PAL1), a longitudinal randomized trial of 999 adolescents and their families recruited in Portland, Oregon. The original study was a National Institute on Drug Abuse funded trial that began in 1996 but was not preregistered. In this study we report secondary analyses on long-term outcomes as part of follow-up assessments to the original study. Primary outcomes of the trial can be found in previous publications^30, 46^. Participants and their families were randomized to intervention and control conditions of the Family Check-Up (FCU) in grade 6^30^. The FCU is designed to reduce adolescent problem behavior by improving parenting and family functioning. The FCU provides strategies focused on building positive parenting skills (e.g., positive reinforcement, limit-setting, problem-solving, communication skills). All adolescents in 6th grade at three middle schools were invited to participate (of which 90% consented). Control participants completed assessments but were not offered intervention services. All methods were carried out in accordance with relevant guidelines and regulations.

Children were initially assessed at 11–12 years old, after which the intervention was administered, followed by four annual assessments (waves 1–5). Additional assessments were administered at 16–17, 18–19, 23–24, 24–25, 26–27, 28–30 years old (waves 6–11) which were used in the present study. Data from waves 1–5 was not applicable as alcohol use was not assessed in the full sample. Retention at the last completed wave of data collection was excellent (83%). The sample was genotyped as part of the age 26–27 assessment. Participants self-reported as 51% male, 44% EA, 30% AA, 13% multiracial, 6% Latino, 4% Asian American, and 4% other groups (Native American, Pacific Islander). The current analyses included participants who had genomic data, alcohol use data across multiple time points, and were either AA (*n* = 192) or EA (*n* = 271) which comprised 46% of the total sample (participants were excluded for the following: 28% were of another ethnicity, 24% were not genotyped; 2% had missing alcohol use data). This sample did not differ from the larger sample based on risk indices, demographic factors, or alcohol use except for higher levels of alcohol use at age 23–24 and 24–25 for those included in the present sample. Intervention condition did not have direct effects on alcohol use, thus, intervention effects were controlled for but were not a focus of the study given the relatively small sample sizes.

### Procedures

All study protocols were approved by the Institutional Review Board of the Oregon Research Institute. Parent or guardian provided written informed consent for all minors and adolescents provided assent for participation in the study, while adult participants provided their own written informed consent.

DNA was collected using the Oragene saliva collection kits in young adulthood (Wave 10) and extracted according to Oragene’s recommended procedures. Genotyping was performed at Rutgers University Cell and DNA Repository (RUCDR) using the Affymetrix BioBank Array. Imputation was conducted to 1000 Genomes (Phase 3 reference panel; 1000 Genomes Project Consortium, 2015) using SHAPEIT2^31^ and then IMPUTE2^32^. The initial imputed data included 39,921,474 SNPs excluding those that failed imputation quality. Of these, SNPs were excluded with a genotyping rate of < 0.95 (n = 37,90,433), that did not pass Hardy–Weinberg equilibrium (HWE; p < 10–6; n = 165,411), or with a minor allele frequency (MAF) < 0.01 (n = 27,840,960). In total, 8,124,670 SNPs passed these quality control and data cleaning thresholds.

### Measures

#### AA and EA PRSs for alcohol use

PRSs were computed based on summary statistics from a multi-ethnic GWAS on alcohol consumption performed separately in EA and AA individuals as part of the Million Veteran Program^29^. In particular, summary statistics were drawn from the discovery GWAS based on EA individuals (N = 200,680) and discovery GWAS based on AA individuals (N = 56,495) on the Alcohol Use Disorder Identification Test-Consumption (AUDIT-C; three items assessing alcohol consumption quantity, frequency, and frequency of heavy drinking) were used in creating polygenic scores. Palindromic SNPs with ambiguous effect directions (A/T or C/G) were removed and AA and EA samples were separately matched with 1000 Genomes and discovery GWAS SNPs resulting in 3,131,481 AA SNPs and 3,247,200 EA SNPs for use in separate PRS-CS calculations. Next, we used PRS-CS to create separate PRSs for each ancestry using different corresponding discovery GWAS weights from the MVP sample. Broadly, the PRS-CS method uses linkage disequilibrium (LD) information from the 1000 Genomes Project European and African reference panels and estimates the posterior effect sizes for SNPs in a given set of GWAS summary statistics. This is different than traditional pruning and thresholdolding PRS methods which use direct beta weights from a discovery GWAS to create several PRS at different statistical thresholds (e.g., PRS at p < 0.0001) which vary by the number of SNPs included in each score. The PRS-CS method^33^ uses a Bayesian regression framework to leverage all available SNPs across the genome, after adjusting for their interdependence, to create a single PRS. Further, the PRS-CS method creates a new set of SNP weights which balances the beta value contribution of each SNP by placing a continuous shrinkage (CS) prior on the SNP effect size from the discovery GWAS. Empirical tests and simulations have shown improved predictive power for PRS-CS scores above traditional methods of polygenic construction. Because PRS-CS uses LD information from an external reference panel, we matched ancestries between the discovery samples and the ancestry reference provided by PRS-CS.

In the current study, we used negative log of the GWAS association *p* value and sign of the association (beta) statistic (-log(*p*-value)*sign(beta)) as initial SNP weights. Initial MVP EA discovery GWAS SNP weights were used for EA individuals in the target sample and, separately, initial MVP AA discovery GWAS weights were used for AA individuals in the target sample. Bayesian regression with the continuous shrinkage method (PRS-CS^33^) was used to create separate PRSs for AA and EA samples. The final PRSs were based on posterior PRS-CS weights and was created using the *score* procedure in PLINK 1.9, averaging by the total number of nonmissing SNPs for each sample^34^ (356,113 SNPs in the final AA PRS; 485,577 SNPs in the final EA PRS).

#### Population stratification and genetic admixture

Principal Components Analysis was conducted to represent population admixture using snpgdsPCA function from R SNPRelate package^35^, after performing LD pruning and filtering using PLINK. The first 20 principal components (PCs) were extracted using 100k SNPS held out for final PC creation. When examining PC eigenvalues and the scree plot, the first two PCs were above the elbow cutoff and explained the greatest variance. The first two PCs were found to reliably distinguish European American, African American, and Latinx/Hispanic ancestry. As a further test in the present data, all 20 PCs were examined relative to alcohol use outcomes using stepwise regression. The first two PCs remained as significantly contributing to these outcomes. Thus, the first two PCs were retained as covariates in analyses.

#### Alcohol use

Alcohol use was assessed at waves 6–11 by separately asking about quantity and frequency of consumption of beer, wine, and hard alcohol (e.g., ‘How often did you drink beer in the last 3 months?’; 0 = Never to 7 = 2–3 times a day or more; ‘How much beer did you drink in the past 3 months?; 0 = Less than one can to 5 = More than 5 cans). Response options were recoded into semicontinuous measures of quantity and frequency of beer, wine, and hard alcohol per month. The two highest response options for frequency of drinking (“once a day” and “2–3 times a day (or more)”) were collapsed into a single value representing daily drinking. Next, we calculated a sum of all quantity items and a sum of all frequency items to obtain monthly measures of quantity and frequency of alcohol use. The monthly measures were then multipled (monthly frequency * monthly quantity) to obtain an overall measure of alcoholic drinks per month at each wave. Mean levels of drinks per month (DPM) varied within and across waves (wave 6 M = 8.21, *SD* = 22.72, *Mdn* = 1.00, *IQR* = 7.00; wave 7 M = 15.02, *SD* = 28.07, *Mdn* = 3.00, *IQR* = 18.13; wave 8 M = 34.30, *SD* = 46.87, *Mdn* = 17.13, *IQR* = 40.38; wave 9 M = 32.92, *SD* = 42.37, *Mdn* = 18.50, *IQR* = 40.50; wave 10 M = 25.71, *SD* = 37.37, *Mdn* = 11.25, *IQR* = 30.50; wave 11 M = 19.87, *SD* = 30.88, *Mdn* = 8.00, *IQR* = 23.00).

#### Covariates

Participant gender, intervention condition, and the first 2 PCs representing genetic ancestry were controlled in all analyses. Given emerging evidence of gene by intervention effects, the interaction between intervention condition and PRS was also controlled^22^.

### Statistical analyses

We examined all relevant statistical assumptions inherent to the application of regression. The PRS variables in the AA and EA samples were normally distributed but measures of alcohol use were positively skewed and kurtotic, which were log transformed. After log-transforming, all alcohol use variables were within recommended limits for skewness and kurtosis (skew: − 0.25 to 0.97, kurtosis: − 1.12 to − 0.07). Mean level differences were tested for alcohol use variables between the AA and EA samples. Mean level differences were not tested for the PRSs as they were based on distinct GWAS which yielded different weights so were uncomparable. TVEM was tested using a time-varying effect modeling macro in SAS v9.4^36^. TVEM is an extension of linear regression but makes no parametric assumptions about the shape (e.g., linear, quadratic) or rate of change over time in associations^20, 36^. Rather, TVEM reflects the shape of change directly from observations by estimating unstandardized regression coefficients and 95% confidence intervals between time-varying predictors and a longitudinal outcome as a function of continuous time (age-based rather than wave-based). In the current study the PRS for alcohol use was considered a time-varying predictor given that the relative influence of polygenic predisposition for alcohol use on alcohol use use may vary during distinct ages. Alcohol use was considered the age-varying outcome. Significant effects are indicated when the 95% confidence interval around a regression coefficient does not include zero. Currently, no formal tests of power exist for TVEM models. Methodological analyses suggest that 100 participants with 10 observations per participant is sufficient for reasonable power, and that with more participants fewer observations are needed^20^. In the current sample, we had six observations (assessed at approximately 17, 19, 22, 23, 28, and 29 years old) but greater sample size in both AA and EA samples indicating the current analyses are likely powered. All analyses were run separately in AA and EA samples to examine for specificity of PRS effects.

## Results

Means, standard deviations, and correlations for the PRSs and alcohol use for the AA and EA samples are presented in Table 1. In the AA sample (above the diagonal), the PRS was positively correlated with alcohol use at age 23–24. In the EA sample (below the diagonal), the PRS was positively correlated with alcohol use at ages 23–24, 26–27, 28–30. Mean level differences between AA and EA samples were detected for alcohol use at each wave (*F*s > 4.72, *p*s < 0.03). The EA sample had higher average alcohol use at each wave compared to the AA sample. Over time alcohol use in both the AA and EA samples peaked at wave 8, however, alcohol use had a lower average peak and decreased more quickly in the AA sample than in the EA sample which had a higher peak and decreased more slowly.

**Table 1:** Correlations, means, and standard deviations among polygenic risk scores and alcohol use for african americans above the diagonal and european americans below the diagonal.

	1	2	3	4	5	6	7	Mean (SD)
1. PRS	1	0.09	0.03	0.18*	0.10	0.09	0.01	− .0.42 (1.06)
2. Alcohol Use Wave 6 (16–17yo)	0.12	1	0.49***	0.16	0.18	0.24*	0.26**	0.29 (0.47)
3. Alcohol Use Wave 7 (18–19yo)	− .0.02	0.31***	1	0.38***	0.37***	0.23**	0.23*	0.50 (0.60)
4. Alcohol Use Wave 8 (23–24yo)	0.14*	0.31***	0.41***	1	0.56***	0.33***	0.41***	1.08 (0.63)
5. Alcohol Use Wave 9 (24–25yo)	0.05	0.28***	0.47***	0.65***	1	0.44***	0.39***	1.06 (0.61)
6. Alcohol Use Wave 10 (26–27yo)	0.13*	0.19**	0.29***	0.46***	0.50***	1	0.60***	0.96 (0.67)
7. Alcohol Use Wave 11 (28–30yo)	0.16*	0.07	0.27***	0.40***	0.45***	0.63***	1	0.78 (0.62)
Mean (SD)	0.35 (0.99)	0.61 (.60)	0.90 (.65)	1.33 (.58)	1.32 (.55)	1.08 (.63)	1.00 (.64)	

*PRS* polygenic risk score. Standardized PRS and log transformed alcohol use scores are presented. *p < .05, **p < .01, ***p < .001.

**Figure 1:**
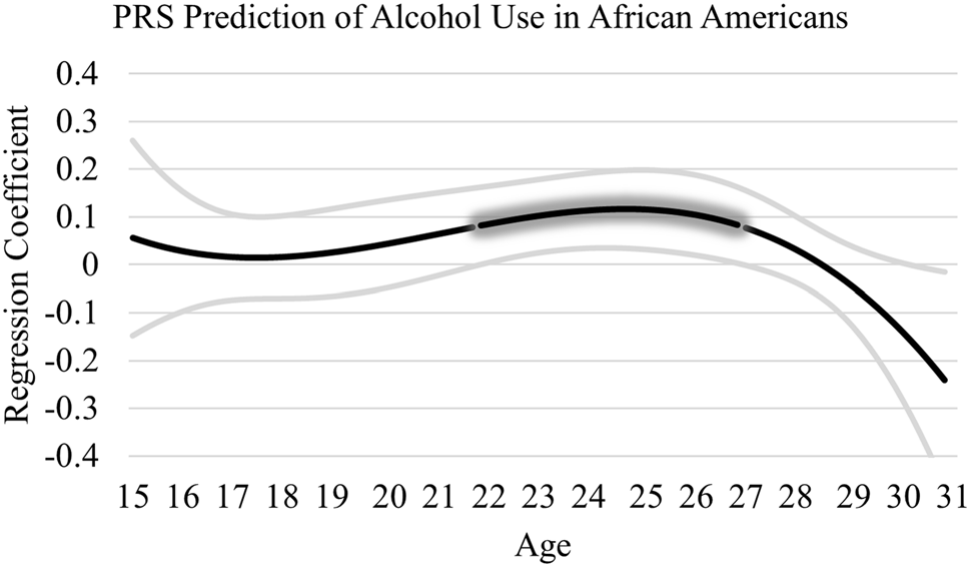
PRS prediction of alcohol use in African Americans. Graph represents age-varying strength of the association between the PRS and alcohol use in African Americans from 15 to 31 years of age. Regression coefficient estimates are represented by the black line and the 95% confidence interval by the gray lines. The “glowing” portion represents a significant association where the 95% confidence interval departs from zero.

**Figure 2:**
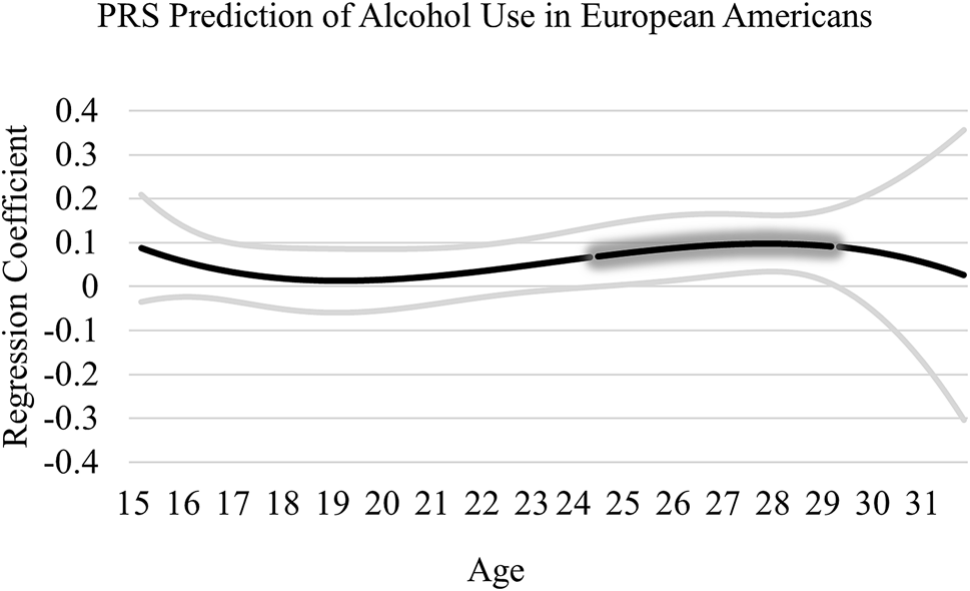
PRS prediction of alcohol use in European Americans. Graph represents age-varying strength of the association between the PRS and alcohol use in European Americans from 15 to 31 years of age. Regression coefficient estimates are represented by the black line and the 95% confidence interval by the gray lines. The “glowing” portion represents a significant association where the 95% confidence interval departs from zero.

TVEM analyses in AA and EA samples were next conducted (see Figs. 1 and 2). In the AA sample, the PRS was associated with alcohol use from approximately age 22 to 27 years of age (association from 22.24 to 27.23 years of age; effect size ranged from 0.08 to 0.12). This effect is illustrated in Fig. 1 by the glowing portion of the line, where the lower bound 95% confidence interval departs from zero. In the EA sample, the PRS was associated with alcohol use from approximately 24.50 to 29 years of age (association from 24.51 to 29.22 years of age; effect size ranged from 0.07 to 0.10), which is presented in Fig. 2. Few covariate effects were detected; male gender was associated with greater alcohol use in both the AA (*B* = -0.03, *p* < 0.001) and EA (-0.04, *p* < 001) samples. The PRS by intervention interaction was not significant in the AA sample (*Z* = 1.40, *p* = 0.16) or EA sample (*Z* = 1.37, *p* = 0.17). No other covariates were associated with alcohol use.

## Discussion

The current study found polygenic risk scores generated from large racially/ethnically-aligned GWAS discovery samples of alcohol use to predict age-varying alcohol use in both AA and EA samples. Broadly, the PRS predicted alcohol use in the AA sample from approximately 22–27 years old and from 24.5–29 in the EA sample. In line with our hypothesis, the PRSs for alcohol use in both AAs and EAs were associated with elevated alcohol use. These findings have important implications for examining developmental genetic effects within specific racial/ethnic populations. Previous genetic studies have identified specific genetic variants influential on alcohol use in early adulthood offering insight into specific developmental effects^11, 37–39^. The current study advances previous work on single candidate genes and research predicting alcohol use at a single distal timepoint^12, 18^. It also extends developmental polygenic research on alcohol use through the use of TVEM to identify developmentally specific genetic influences.

The developmental course of alcohol use for both AA and EA samples was in line with typical patterns of alcohol use across adolescence and early adulthood^1^. The PRS appeared to capture peak alcohol use. In the AA sample, the PRS was first associated with alcohol use in early adulthood at age 22, then increased in a curvilinear fashion to age 27 when there was a slight decline in the effect. During this period, mean levels of alcohol use peaked at approximately the beginning of this age span and declined steadily throughout, consistent with the genetic effect, until dropping off sharply in middle adulthood when the genetic effect was absent (wave 11: 28–30 years old). In the EA sample the PRS was associated with alcohol use from age 24.50 to 29. During this period, mean levels of alcohol use peaked at the beginning of this age span and remained elevated, eventually declining, which likely contributed to the extended genetic effect to age 29 but no apparent decline in the effect. The specificity of the present findings to early adulthood may be for a number of reasons.

Theoretically, alcohol *initiation* in adolescence is primarily influenced by environmental factors, such as affiliation with deviant peers, but genetic influences underlie increases in alcohol *use* and more stable patterns of drinking in early to middle adulthood^1, 11^. The GWAS leveraged in the current study was on alcohol use, and not misuse, in an older population so it is likely that the genetic signal captured in our PRS reflects elevated alcohol use irrespective of age. In the current sample this is reflected by PRS associations with alcohol use during early adulthood, when there was elevated alcohol use in both AA and EA samples. It is likely that the present PRS for alcohol use is capturing a portion of the genetic effect related to elevated or increasing alcohol use in early adulthood for both AAs and EAs. Accordingly, previous candidate gene studies have identified specific variants associated with alcohol use in early adulthood, including *GABRA2*^37^, *5HTT*^39^ and *ALDH2*^38^. Theoretically, biological and socially mediated mechanisms may explain genetic effects underlying alcohol use^11^. For example, with age individuals have a greater ability to seek or select social environments conducive to alcohol use, and this behavior may be driven in part by a genetic predisposition for alcohol use. This may be especially true as individuals reach legal drinking age in early adulthood and thereafter have greater access and freedom to use alcohol. Thus, elevated drinking during this period may reflect a genetic influence. Given the novelty of TVEM, and relatively small effect sizes, replication of these findings is an important endeavor for future research.

Complentary to this, the declining effect in AAs and absence of genetic effects with age in AAs and EAs may be due to a number of reasons. As individuals progress into adulthood, there are often developmental reductions in alcohol use as individuals transition to full-time jobs and raise families^6^. Such environmental factors may account for the decreasing genetic influence seen in adulthood in the present study. Although we did observe reductions in alcohol use with age our study is mute regarding environmental processes involved in alcohol use during this period which may differ for AA and EA individuals. Conversely, the attenuation of genetic effects could also be explained by a sparsity of data points at greater ages. To note, effect sizes identified in TVEM and the zero-order correlations were small in size, which is in line with previous polygenic research.

The current findings regarding mean level differences in alcohol use in AAs and EAs are consistent with previous literature indiciating less alcohol use frequency, quantity, and misuse in AAs compared to EAs^7, 8^; and with research indicating that across adolescence, EAs alcohol use increases more sharply and remains elevated for longer periods^40^. Both these findings were supported in the present study as EAs had greater average alcohol use than AAs at each wave, and also had greater peak usage that declined more slowly. This may be the case for a number of reasons. Research indicates that for AAs, compared to EAs, alcohol use can be less culturally engrained^41^, with more norms against heavy drinking^42^. Further, although AAs consume less alcohol than EAs, they are more likely to face societal consequences when consuming alcohol, leading to less consumption but more negative repercussions when consuming, such as legal issues and resulting greater risk for alcohol abuse, dependence, and addiction^43^. This may occur, in part, because AAs experiences greater contextual risk factors for developing alcohol use problems including discrimination, residential segregation, and limited access to adequate health resources^5, 43^. This disparity makes identifying etiological influences underlying alcohol use across multiple time points and in diverse populations an important endeavor.

Collectively, these findings are highlighted by a number of strengths including the use of multi-ethnic PRSs based on large multi-ethnic GWASs and the use of TVEM as a developmentally sensitive method. This approach moves towards more accurately characterizing genetic predisposition and genetic effects in multi-ethnic populations. Given the novelty of this approach, this resulted in differences in the sample sizes of the discovery GWAS and in the number of SNPs that went into the AA and EA PRSs. Thus, it is important that future studies pursue replication of these findings. We made multiple efforts to attempt replication of these findings but were unable to find a suitable sample despite searching multiple independent worldwide datasets given the unique characteristics of the present sample and specific longitudinal data requirements (ethnically diverse, genotyped, adequate sample size, longitudinal alcohol measurement at multiple time points). Future studies could be aided by genotyping existing richly measured longitudinal samples.

In addition, it should be noted that participants in the present sample participated in a preventative intervention during adolescence. No difference in alcohol use were detected in preliminary analyses, or using TVEM for individuals based on intervention vs. control conditions, or for the PRS by intervention effect on alcohol use. However, the intervention may have affected intermediary processes such as parenting, the family environment, or youth attitudes about alcohol use. Indeed, in the same sample the FCU has shown to reduce family conflict, antisocial behavior, and involvement with deviant peers^44^. Further, the FCU has shown some prevention effects on trajectories of substance use in adolescence^45, 46^, but this has not been investigated later in life. Thus, there may be indirect effects on participant’s alcohol use which were outside the scope of the present study and should be examined in future research.

Limitations should be mentioned. The respective AA and EA samples had relatively small sample sizes which are in line with power recommendations for TVEM^20^, but warrant replication. Further, at present it is not possible to test for differences across models using TVEM or measures of effect size but significant mean level differences in AA and EA alcohol provide preliminary evidence for differential patterns of drinking. Effect sizes from the zero-order correlations indicated small effect sizes which are in accord with previous polygenic research. Also, the discovery GWAS sample was older (majority 50 to 70 years old) compared to the current sample so we were unable to examine associations during the same developmental period. Relatedly, regular alcohol use in older age (as in the discovery GWAS) is normative and substantively different than during adolescence when alcohol use first emerges and there is typically lower levels of use. This may have contributed to a lack of associations prior to regular use in early adulthood. A future goal for polygenic research is aligning developmental *and* racial/ethnic characteristics in discovery and target samples to identify genetic signals specific to developmental periods and specific racial/ethnic populations. Finally, we were unable to replicate findings in a independent dataset given the unique characteristics of the present sample. Despite these limitations, these results replicate genotype–phenotype associations in AA and EA samples that overlap but are developmentally distinct. This extends current developmental alcohol literature as well as genetic association research in multiple racial/ethnic samples. Future research should replicate findings in independent samples. Our findings also highlight the need for larger GWAS in ethnically and developmentally diverse samples to facilitate examination of these processes in different populations.

This research highlights the importance of including diverse populations in genetic research^47, 48^. It also has important practical implications, such as illustrating that genetic predisposition for alcohol use can vary developmentally based on racial/ethnic characteristics of the sample. This can help guide tailored intervention approaches; preventive intervention programs known to decrease the development of alcohol use disorders and related problems could be administered based on the intersection of age and demographic characteristics. Additonal strategies could focus on buffering social influences but also individual characteristics reflective of genetic predisposition. For instance, recent approaches are focusing on moving away from a focus on substance use to identification of genetically-based underlying risk factors, such as behavioral dishinhibition and externalizing^49^. Such approaches could identify those at-risk for developing alcohol use problems during developmentally sensitive periods.

## Data Availability

Data from the current study is available upon reasonable request from the principal investigator of the Project Alliance 1 study, Thao Ha (thaoha@asu.edu).
